# 5-(3,4-Dimeth­oxy­benzyl­idene)-1,3-dimethyl-1,3-diazinane-2,4,6-trione

**DOI:** 10.1107/S1600536811052986

**Published:** 2011-12-21

**Authors:** Mukut Gohain, Theunis J. Muller, Barend C. B. Bezuidenhoudt

**Affiliations:** a Department of Chemistry, University of the Free State, PO Box 339, Bloemfontein 9300, South Africa

## Abstract

In the title compound, C_15_H_16_N_2_O_5_, the dihedral angle between 1,3-diazinane and benzene rings is only 4.27 (1)°. The essentially planar mol­ecular structure is characterized by a short intra­molecular C—H⋯O separation and by an exceptionally large bond angle of 138.25 (14)° at the bridging methine C atom. The meth­oxy groups deviate somewhat from the plane of the benzene ring, with C—C—O—C torsion angles of −15.6 (1) and 9.17 (6)°. In the crystal, mol­ecules form centrosymmetric dimers *via* donor–acceptor π–π inter­actions, with a centroid–centroid distance of 3.401 (1) Å.

## Related literature

For the biological activity of 1,3-diazinane derivatives, see: Negwar (2001 ▶); Tanaka *et al.* (1986 ▶, 1988 ▶). For the use of pyridine-type ligands in catalysis models, see: Roodt *et al.* (2011 ▶); van der Westhuizen *et al.* (2010 ▶). For related structures, see: Panchatcharam *et al.* (2009 ▶); Rezende *et al.* (2005 ▶). For the synthesis, see: Prajapati *et al.* (2006 ▶). For standard bond lengths, see: Allen *et al.* (1987 ▶).
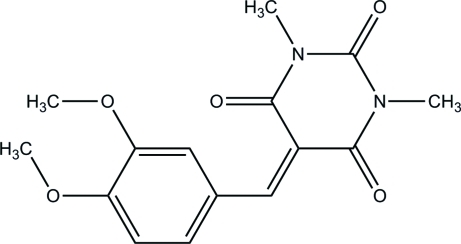


## Experimental

### 

#### Crystal data

C_15_H_16_N_2_O_5_
*M* *_r_* = 304.3
Triclinic, 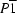

*a* = 7.3086 (2) Å
*b* = 8.4033 (3) Å
*c* = 11.8705 (5) Å
α = 82.5685 (18)°
β = 77.6686 (17)°
γ = 71.1469 (15)°
*V* = 672.58 (4) Å^3^
*Z* = 2
Mo *K*α radiation
μ = 0.11 mm^−1^
*T* = 100 K
0.15 × 0.12 × 0.06 mm

#### Data collection

Bruker APEXII CCD diffractometer
Absorption correction: multi-scan (*SADABS*; Bruker, 2008 ▶) *T*_min_ = 0.984, *T*_max_ = 0.994
12172 measured reflections
3233 independent reflections
2478 reflections with *I* > 2σ(*I*)
*R*_int_ = 0.032

#### Refinement

*R*[*F* ^2^ > 2σ(*F* ^2^)] = 0.042
*wR*(*F* ^2^) = 0.113
*S* = 1.05
3233 reflections
203 parameters
H-atom parameters constrained
Δρ_max_ = 0.32 e Å^−3^
Δρ_min_ = −0.30 e Å^−3^

### 

Data collection: *APEX2* (Bruker, 2008 ▶); cell refinement: *SAINT-Plus* (Bruker, 2008 ▶); data reduction: *SAINT-Plus*; program(s) used to solve structure: *SHELXS97* (Sheldrick, 2008 ▶); program(s) used to refine structure: *SHELXL97* (Sheldrick, 2008 ▶); molecular graphics: *DIAMOND* (Brandenberg & Putz, 2005 ▶); software used to prepare material for publication: *WinGX* (Farrugia, 1999 ▶).

## Supplementary Material

Crystal structure: contains datablock(s) global, I. DOI: 10.1107/S1600536811052986/ld2039sup1.cif

Structure factors: contains datablock(s) I. DOI: 10.1107/S1600536811052986/ld2039Isup2.hkl

Supplementary material file. DOI: 10.1107/S1600536811052986/ld2039Isup3.cml

Additional supplementary materials:  crystallographic information; 3D view; checkCIF report

## Figures and Tables

**Table 1:** Hydrogen-bond geometry (Å, °)

*D*—H⋯*A*	*D*—H	H⋯*A*	*D*⋯*A*	*D*—H⋯*A*
C7—H7⋯O3	0.93	2.08	2.871 (2)	142

**Fig. 1.**
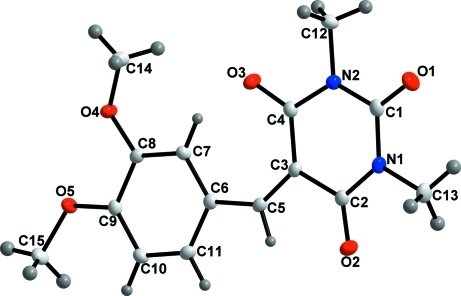
Diamond representation of the title compound, showing the numbering scheme and displacement ellipsoids (50% probability).

**Fig. 2.**
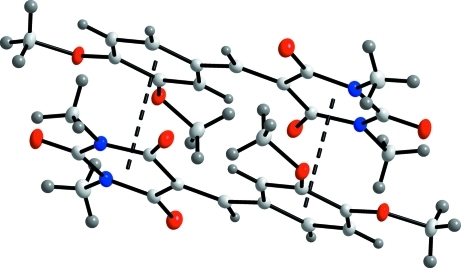
Diamond representation of the title compound, showing the π-π interaction.

## supplementary crystallographic information

### Comment

Barbituric acid is the parent compound of barbiturate drugs, although by itself it is not pharmacologically active (Negwar *et al.*, 2001). Benzyledenebarbituric acids are important building block in the synthesis of pyrazolo-[3,4]-1,3-diazinane derivatives which shows broad-spectrum biological activities (Tanaka *et al.*, 1986 and 1988). We also synthesized some of the benzyledene barbituric acids which were successfully used to prepare pyrano[2,3-*d*]- and furopyrano[2,3-*d*] 1,3-diazinane derivatives (Prajapati *et al.*, 2006). The title compound having molecular formula C_15_H_16_N_2_O_5_ can be prepared by the condensation of barbituric acid and 4,5-dimethoxybenzaldehyde. The bond C5—C6 of 1.453 (2) Å is longer than C3—C5 bond of 1.365 (2) Å that indicates C3—C5 as a formally double bond. This is in accordance with the literature (Panchatcharam *et al.* 2009 and Rezende *et al.* 2005).

### Experimental

Mixture of *N*,*N*-dimethylbarbituric acid (0.50 g, 3.2 mmol) and 4,5-dimethoxy benzaldehyde (0.53 g, 3.2 mmol) in ethanol (10 ml) was stirred at room temperature until completion of the reaction (monitored by TLC). The solids that precipitated during the course of the reaction were filtered and washed with diethyl ether (5 ml). The precipitate was subsequently dissolved in hot acetonitrile. Upon cooling to room temperature with a slow evaporation of the acetonitrile the crystals (mp 229–230 °C) suitable for single-crystal X-ray diffraction were obtained.

^1^H NMR (600 MHz): 3.42 (s, 3H, N—Me), 3.43 (s, 3H, N—Me), 3.99 (s, 3H, OMe), 3.40 (s, 3H, OMe), 6.97 (d, 1H), 7.81 (dd, 1H), 8.41 (d, 1H), 8.51(s, 1H).

^13^C {^1^H} NMR (150Mz): 28.5, 29.1, 56.1, 56.2, 110.4, 114.2, 116.6, 125.9, 132.6, 148.4, 151.4, 154.4, 159.2, 161.1, 163.3.

### Refinement

The aromatic H atoms were positioned geometrically and allowed to ride on their parent atoms, with *U*_iso_(H) = 1.2*U*_eq_(parent) of the parent atom with a C—H distance of 0.93. The methyl H atoms were placed in geometrically idealized positions and constrained to ride on its parent atoms with *U*_iso_(H) = 1.5*U*_eq_(C) and at a distance of 0.96 Å; their torsion angles were optimized from electron density

### Crystal data

**Table:**

C_15_H_16_N_2_O_5_	*Z* = 2
*M**_r_* = 304.3	*F*(000) = 320
Triclinic, *P*1	*D*_x_ = 1.508 Mg m^−^^3^
Hall symbol: -P 1	Mo *K*α radiation, λ = 0.71073 Å
*a* = 7.3086 (2) Å	Cell parameters from 3146 reflections
*b* = 8.4033 (3) Å	θ = 2.6–28.2°
*c* = 11.8705 (5) Å	µ = 0.11 mm^−^^1^
α = 82.5685 (18)°	*T* = 100 K
β = 77.6686 (17)°	Plate, yellow
γ = 71.1469 (15)°	0.15 × 0.12 × 0.06 mm
*V* = 672.58 (4) Å^3^	

### Data collection

**Table:**

Bruker APEXII CCD diffractometer	2478 reflections with *I* > 2σ(*I*)
graphite	*R*_int_ = 0.032
φ and ω scans	θ_max_ = 28°, θ_min_ = 3.2°
Absorption correction: multi-scan (*SADABS*; Bruker, 2008)	*h* = −9→9
*T*_min_ = 0.984, *T*_max_ = 0.994	*k* = −11→11
12172 measured reflections	*l* = −15→15
3233 independent reflections	

### Refinement

**Table:**

Refinement on *F*^2^	Primary atom site location: structure-invariant direct methods
Least-squares matrix: full	Secondary atom site location: difference Fourier map
*R*[*F*^2^ > 2σ(*F*^2^)] = 0.042	Hydrogen site location: inferred from neighbouring sites
*wR*(*F*^2^) = 0.113	H-atom parameters constrained
*S* = 1.05	*w* = 1/[σ^2^(*F*_o_^2^) + (0.0512*P*)^2^ + 0.239*P*] where *P* = (*F*_o_^2^ + 2*F*_c_^2^)/3
3233 reflections	(Δ/σ)_max_ = 0.003
203 parameters	Δρ_max_ = 0.32 e Å^−^^3^
0 restraints	Δρ_min_ = −0.30 e Å^−^^3^

### Special details

**Table:**

Experimental. The intensity data was collected on a Bruker X8 ApexII 4 K Kappa CCD diffractometer using an exposure time of 15 s/frame. A total of 1821 frames were collected with a frame width of 0.5° covering up to θ = 28.18° with 99.7% completeness accomplished.
Geometry. All s.u.'s (except the s.u. in the dihedral angle between two l.s. planes) are estimated using the full covariance matrix. The cell s.u.'s are taken into account individually in the estimation of s.u.'s in distances, angles and torsion angles; correlations between s.u.'s in cell parameters are only used when they are defined by crystal symmetry. An approximate (isotropic) treatment of cell s.u.'s is used for estimating s.u.'s involving l.s. planes.
Refinement. Refinement of *F*^2^ against ALL reflections. The weighted *R*-factor *wR* and goodness of fit *S* are based on *F*^2^, conventional *R*-factors *R* are based on *F*, with *F* set to zero for negative *F*^2^. The threshold expression of *F*^2^ > 2σ(*F*^2^) is used only for calculating *R*-factors(gt) *etc*. and is not relevant to the choice of reflections for refinement. *R*-factors based on *F*^2^ are statistically about twice as large as those based on *F*, and *R*- factors based on ALL data will be even larger.

### Fractional atomic coordinates and isotropic or equivalent isotropic displacement parameters (Å^2^)

**Table:**

	*x*	*y*	*z*	*U*_iso_*/*U*_eq_	
C1	0.4027 (2)	0.72599 (19)	0.80997 (12)	0.0160 (3)	
C2	0.5148 (2)	0.43533 (18)	0.75097 (12)	0.0150 (3)	
C3	0.61438 (19)	0.49161 (18)	0.63713 (12)	0.0135 (3)	
C4	0.59797 (19)	0.67081 (18)	0.61360 (12)	0.0144 (3)	
C5	0.70824 (19)	0.36478 (18)	0.56448 (12)	0.0139 (3)	
H5	0.6895	0.2635	0.5979	0.017*	
C6	0.82904 (19)	0.34247 (18)	0.44990 (12)	0.0138 (3)	
C7	0.89371 (19)	0.46673 (18)	0.37541 (12)	0.0142 (3)	
H7	0.8572	0.576	0.3985	0.017*	
C8	1.01025 (19)	0.42763 (18)	0.26903 (12)	0.0138 (3)	
C9	1.06655 (19)	0.26239 (18)	0.23180 (12)	0.0142 (3)	
C10	1.0028 (2)	0.13956 (18)	0.30414 (12)	0.0160 (3)	
H10	1.0382	0.0306	0.2807	0.019*	
C11	0.8865 (2)	0.17974 (18)	0.41127 (12)	0.0155 (3)	
H11	0.8452	0.0963	0.459	0.019*	
C12	0.4873 (2)	0.95348 (18)	0.68221 (13)	0.0186 (3)	
H12A	0.3864	1.0081	0.6375	0.028*	
H12B	0.6109	0.9644	0.6403	0.028*	
H12C	0.4551	1.0052	0.7544	0.028*	
C13	0.3059 (2)	0.5045 (2)	0.93915 (13)	0.0215 (3)	
H13A	0.2832	0.5852	0.9949	0.032*	
H13B	0.3826	0.396	0.9664	0.032*	
H13C	0.1822	0.498	0.9283	0.032*	
C14	0.9864 (2)	0.71432 (18)	0.21141 (14)	0.0203 (3)	
H14A	0.8468	0.7402	0.2177	0.03*	
H14B	1.0356	0.7815	0.1479	0.03*	
H14C	1.0144	0.7384	0.2817	0.03*	
C15	1.2176 (2)	0.08217 (19)	0.07552 (14)	0.0215 (3)	
H15A	1.29	−0.0087	0.122	0.032*	
H15B	1.2928	0.0848	−0.0012	0.032*	
H15C	1.0949	0.0657	0.0724	0.032*	
N1	0.41279 (17)	0.55732 (16)	0.82856 (10)	0.0161 (3)	
N2	0.50181 (17)	0.77434 (15)	0.70439 (10)	0.0151 (3)	
O5	1.18050 (15)	0.23929 (13)	0.12565 (9)	0.0181 (2)	
O1	0.31031 (16)	0.82697 (14)	0.88192 (9)	0.0229 (3)	
O2	0.51855 (15)	0.28912 (13)	0.77753 (9)	0.0216 (3)	
O3	0.66023 (15)	0.73491 (13)	0.52101 (9)	0.0210 (3)	
O4	1.07951 (14)	0.53932 (13)	0.19183 (9)	0.0174 (2)	

### Atomic displacement parameters (Å^2^)

**Table:**

	*U*^11^	*U*^22^	*U*^33^	*U*^12^	*U*^13^	*U*^23^
C1	0.0140 (6)	0.0205 (8)	0.0140 (7)	−0.0057 (6)	−0.0022 (5)	−0.0026 (6)
C2	0.0151 (6)	0.0175 (7)	0.0130 (7)	−0.0061 (6)	−0.0020 (5)	−0.0008 (6)
C3	0.0134 (6)	0.0152 (7)	0.0116 (7)	−0.0049 (5)	−0.0022 (5)	0.0006 (5)
C4	0.0129 (6)	0.0161 (7)	0.0135 (7)	−0.0035 (5)	−0.0013 (5)	−0.0022 (6)
C5	0.0138 (6)	0.0149 (7)	0.0135 (7)	−0.0058 (5)	−0.0027 (5)	0.0013 (6)
C6	0.0128 (6)	0.0156 (7)	0.0128 (7)	−0.0041 (5)	−0.0024 (5)	−0.0010 (5)
C7	0.0148 (6)	0.0137 (7)	0.0141 (7)	−0.0048 (5)	−0.0006 (5)	−0.0028 (5)
C8	0.0136 (6)	0.0147 (7)	0.0134 (7)	−0.0061 (5)	−0.0014 (5)	0.0009 (5)
C9	0.0134 (6)	0.0164 (7)	0.0124 (7)	−0.0042 (5)	−0.0015 (5)	−0.0023 (6)
C10	0.0172 (7)	0.0137 (7)	0.0167 (7)	−0.0040 (5)	−0.0024 (5)	−0.0032 (6)
C11	0.0161 (6)	0.0151 (7)	0.0160 (7)	−0.0070 (6)	−0.0023 (5)	0.0012 (6)
C12	0.0216 (7)	0.0140 (7)	0.0201 (8)	−0.0058 (6)	−0.0013 (6)	−0.0034 (6)
C13	0.0228 (8)	0.0279 (9)	0.0138 (8)	−0.0110 (7)	0.0020 (6)	−0.0015 (6)
C14	0.0237 (7)	0.0153 (8)	0.0203 (8)	−0.0067 (6)	0.0005 (6)	−0.0010 (6)
C15	0.0267 (8)	0.0176 (8)	0.0184 (8)	−0.0053 (6)	0.0009 (6)	−0.0067 (6)
N1	0.0171 (6)	0.0192 (7)	0.0118 (6)	−0.0075 (5)	0.0008 (5)	−0.0008 (5)
N2	0.0164 (6)	0.0144 (6)	0.0143 (6)	−0.0055 (5)	−0.0007 (5)	−0.0013 (5)
O5	0.0226 (5)	0.0156 (5)	0.0142 (5)	−0.0063 (4)	0.0041 (4)	−0.0049 (4)
O1	0.0253 (6)	0.0234 (6)	0.0184 (6)	−0.0077 (5)	0.0040 (4)	−0.0085 (5)
O2	0.0284 (6)	0.0173 (6)	0.0177 (6)	−0.0099 (5)	0.0016 (4)	0.0015 (4)
O3	0.0271 (6)	0.0154 (5)	0.0160 (6)	−0.0056 (4)	0.0031 (4)	0.0008 (4)
O4	0.0214 (5)	0.0133 (5)	0.0154 (5)	−0.0067 (4)	0.0033 (4)	−0.0008 (4)

### Geometric parameters (Å, °)

**Table:**

C1—O1	1.2150 (18)	C10—C11	1.385 (2)
C1—N1	1.386 (2)	C10—H10	0.93
C1—N2	1.3897 (19)	C11—H11	0.93
C2—O2	1.2212 (19)	C12—N2	1.467 (2)
C2—N1	1.3826 (19)	C12—H12A	0.96
C2—C3	1.489 (2)	C12—H12B	0.96
C3—C5	1.365 (2)	C12—H12C	0.96
C3—C4	1.466 (2)	C13—N1	1.4716 (19)
C4—O3	1.2235 (17)	C13—H13A	0.96
C4—N2	1.3914 (19)	C13—H13B	0.96
C5—C6	1.453 (2)	C13—H13C	0.96
C5—H5	0.93	C14—O4	1.4331 (19)
C6—C11	1.402 (2)	C14—H14A	0.96
C6—C7	1.413 (2)	C14—H14B	0.96
C7—C8	1.377 (2)	C14—H14C	0.96
C7—H7	0.93	C15—O5	1.4400 (19)
C8—O4	1.3650 (18)	C15—H15A	0.96
C8—C9	1.416 (2)	C15—H15B	0.96
C9—O5	1.3525 (17)	C15—H15C	0.96
C9—C10	1.389 (2)		
			
O1—C1—N1	121.71 (14)	N2—C12—H12A	109.5
O1—C1—N2	121.60 (14)	N2—C12—H12B	109.5
N1—C1—N2	116.69 (12)	H12A—C12—H12B	109.5
O2—C2—N1	119.59 (13)	N2—C12—H12C	109.5
O2—C2—C3	123.24 (13)	H12A—C12—H12C	109.5
N1—C2—C3	117.16 (13)	H12B—C12—H12C	109.5
C5—C3—C4	127.56 (13)	N1—C13—H13A	109.5
C5—C3—C2	113.69 (13)	N1—C13—H13B	109.5
C4—C3—C2	118.73 (12)	H13A—C13—H13B	109.5
O3—C4—N2	118.37 (14)	N1—C13—H13C	109.5
O3—C4—C3	125.11 (13)	H13A—C13—H13C	109.5
N2—C4—C3	116.51 (13)	H13B—C13—H13C	109.5
C3—C5—C6	138.25 (14)	O4—C14—H14A	109.5
C3—C5—H5	110.9	O4—C14—H14B	109.5
C6—C5—H5	110.9	H14A—C14—H14B	109.5
C11—C6—C7	117.75 (13)	O4—C14—H14C	109.5
C11—C6—C5	115.55 (13)	H14A—C14—H14C	109.5
C7—C6—C5	126.71 (13)	H14B—C14—H14C	109.5
C8—C7—C6	120.66 (13)	O5—C15—H15A	109.5
C8—C7—H7	119.7	O5—C15—H15B	109.5
C6—C7—H7	119.7	H15A—C15—H15B	109.5
O4—C8—C7	124.60 (13)	O5—C15—H15C	109.5
O4—C8—C9	114.77 (12)	H15A—C15—H15C	109.5
C7—C8—C9	120.64 (13)	H15B—C15—H15C	109.5
O5—C9—C10	125.58 (13)	C2—N1—C1	124.99 (13)
O5—C9—C8	115.28 (12)	C2—N1—C13	117.62 (13)
C10—C9—C8	119.14 (13)	C1—N1—C13	117.39 (12)
C11—C10—C9	119.85 (14)	C1—N2—C4	125.56 (13)
C11—C10—H10	120.1	C1—N2—C12	117.23 (12)
C9—C10—H10	120.1	C4—N2—C12	116.91 (12)
C10—C11—C6	121.96 (13)	C9—O5—C15	117.89 (11)
C10—C11—H11	119	C8—O4—C14	116.37 (11)
C6—C11—H11	119		

### Hydrogen-bond geometry (Å, °)

**Table:**

*D*—H···*A*	*D*—H	H···*A*	*D*···*A*	*D*—H···*A*
C7—H7···O3	0.93	2.08	2.871 (2)	142.

## References

[bb1] Allen, F. H., Kennard, O., Watson, D. G., Brammer, L., Orpen, A. G. & Taylor, R. (1987). *J. Chem. Soc. Perkin Trans. 2*, pp. S1–19.

[bb2] Brandenberg, K. & Putz, H. (2005). *DIAMOND* Crystal Impact, Bonn, Germany.

[bb3] Bruker (2008). *APEX2*, *SAINT-Plus* and *SADABS* Bruker AXS Inc., Madison, Wisconsin, USA.

[bb4] Farrugia, L. J. (1999). *J. Appl. Cryst.* **32**, 837–838.

[bb5] Negwar, M. (2001). *Organic–Chemical Drugs and their Synonyms*, 7th Rev. and Engl. ed., Vol. 4, pp. 2873–2957. Berlin: Akademie.

[bb6] Panchatcharam, R., Dhayalan, V., Mohanakrishnan, A. K., Chakkaravarthi, G. & Manivannan, V. (2009). *Acta Cryst.* E**65**, o2394. 10.1107/S1600536809035521 PMC2970469 21577857

[bb7] Prajapati, D. & Gohain, M. (2006). *Beilstein J. Org. Chem.* **2**, No. 11, doi:10.1186/1860-5397-2-11. 10.1186/1860-5397-2-11 PMC1525172 16768808

[bb8] Rezende, M. C., Dominguez, M., Wardell, J. L., Skakle, J. M. S., Low, J. N. & Glidewell, C. (2005). *Acta Cryst.* C**61**, o306–o311. 10.1107/S0108270105008498 15876723

[bb9] Roodt, A., Visser, H. G. & Brink, A. (2011). *Crystallogr. Rev.* **66**, 241–280.

[bb10] Sheldrick, G. M. (2008). *Acta Cryst.* A**64**, 112–122. 10.1107/S0108767307043930 18156677

[bb11] Tanaka, K., Chen, X., Kimura, T. & Yoneda, F. (1986). *Chem. Pharm. Bull.* **34**, 3945–3948.

[bb12] Tanaka, K., Chen, X., Kimura, T. & Yoneda, F. (1988). *Chem. Pharm. Bull.* **36**, 66–69.

[bb13] Westhuizen, H. J. van der, Meijboom, R., Schutte, M. & Roodt, A. (2010). *Inorg. Chem.* **49**, 9599–9608. 10.1021/ic101274q 20836511

